# Role of Nutrition in Prevention of Neonatal Spontaneous Intestinal Perforation and Its Complications: A Systematic Review

**DOI:** 10.3390/nu12051347

**Published:** 2020-05-08

**Authors:** Oluwabunmi Olaloye, Matthew Swatski, Liza Konnikova

**Affiliations:** 1 Division of Newborn Medicine, University of Pittsburgh Medical Center Children’s Hospital of Pittsburgh, Pittsburgh, PA 15224, USA; olaloyeoo@upmc.edu; 2 Department of Pediatrics, Yale Medical School, New Haven, CT 06520, USA; 3 School of Medicine, University of Pittsburgh, Pittsburgh, PA 15213, USA; swatski.matthew@medstudent.pitt.edu; 4 Department of Immunology, University of Pittsburgh, Pittsburgh, PA 15213, USA; 5 Department of Developmental Biology, University of Pittsburgh, Pittsburgh, PA 15213, USA

**Keywords:** spontaneous intestinal perforation, prematurity, feeding, nutrition

## Abstract

Background: Spontaneous intestinal perforation (SIP) is a devastating complication of prematurity, and extremely low birthweight (ELBW < 1000 g) infants born prior to 28 weeks are at highest risk. The role of nutrition and feeding practices in prevention and complications of SIP is unclear. The purpose of this review is to compile evidence to support early nutrition initiation in infants at risk for and after surgery for SIP. ***Methods***: A search of PubMed, EMBASE and Medline was performed using relevant search terms according to Preferred Reporting Items for Systematic Reviews and Meta-Analyses (PRISMA) guidelines. Abstracts and full texts were reviewed by co-first authors. Studies with infants diagnosed with SIP that included information on nutrition/feeding practices prior to SIP and post-operatively were included. Primary outcome was time to first feed. Secondary outcomes were incidence of SIP, time to full enteral feeds, duration of parenteral nutrition, length of stay, neurodevelopmental outcomes and mortality. ***Results***: Nineteen articles met inclusion criteria—nine studies included feeding/nutrition data prior to SIP and ten studies included data on post-operative nutrition. Two case series, one cohort study and sixteen historical control studies were included. Three studies showed reduced incidence of SIP with initiation of enteral nutrition in the first three days of life. Two studies showed reduced mortality and neurodevelopmental impairment in infants with early feeding. ***Conclusions***: Available data suggest that early enteral nutrition in ELBW infants reduces incidence of SIP without increased mortality.

## 1. Introduction

Spontaneous intestinal perforation (SIP) is a devastating gastrointestinal complication of prematurity that occurs within the first week of life in infants born prior to 28 weeks of gestational age (GA) and with extremely low birthweight (ELBW < 1000 g) [1]. The incidence of SIP is highest in the most vulnerable preterm infants [2,3] with high frequency of long-term complications and high economic burden. Necrotizing enterocolitis (NEC), another gastrointestinal complication of prematurity that occurs slightly later, is a separate clinical entity. Both NEC and SIP can present with abdominal distension, temperature and hemodynamic instability [4,5]. NEC is distinguished by the presence of a thickened abdominal wall, distended loops and presence of pneumatosis intestinalis [5], while more patients with SIP present with a bluish discoloration of the abdominal wall and pneumoperitoneum on radiographs [5]. Infants with SIP typically present with isolated intestinal perforation diagnosed as free abdominal air [6,7] and on histology there is evidence of hemorrhagic necrosis primarily in the antimesenteric border of the terminal ileum [5]. On the contrary, NEC is characterized by severe inflammation and bacterial translocation resulting in intraluminal air and intestinal perforation in severe cases [8]. 

Currently, SIP is thought to be secondary to ischemia [9,10] and involves a deficiency of muscularis propria in about a quarter of cases [11]. SIP often occurs in the terminal ileum, a watershed region prone to local ischemia that can be compounded by regional intestinal ischemia, secondary to hypotension, the presence of an umbilical arterial catheter (UAC), patent ductus arteriosus (PDA) and birth asphyxia [9,12]. Local ischemia, impaired collagen synthesis from early steroid use, birth trauma and abnormal embryologic development can result in muscularis propria deficiency that can similarly lead to SIP [10,13,14]. Likewise, antenatal and postnatal factors (outlined in Table 1) can increase the risk of SIP occurrence in infants at greatest risk.

An understanding of preventative strategies for developing SIP is critical as early complications such as intestinal failure [15] can be severe, resulting in a prolonged neonatal intensive care unit (NICU) stay and long-term complications [16,17,18]. Similarly, data on any protective factors that are crucial in SIP prevention are limited. While there is extensive data on the relationship between early nutrition and the incidence of NEC [19,20], studies on feeding practices prior to and after the development of SIP are limited. Given the high morbidity and mortality related to SIP, insight into risk factor modification, specifically nutrition, is essential. We sought to systematically identify and review literature on early feeding prior to and after surgery for SIP to assess safety and potential benefits. 

## 2. Materials and Methods

This review was conducted according to the Preferred Reporting Items for Systematic Reviews and Meta-Analyses (PRISMA) guidelines.

### 2.1. Search Strategy

An electronic search of online databases—PubMed, Medlin and Embase—was conducted January to March 2020 using the following search terms: “spontaneous intestinal perforation,” “neonate,” “newborn” and “nutrition.” Reference lists from resulting articles were also reviewed for additional studies.

### 2.2. Inclusion and Exclusion Criteria

**Figure 1:**
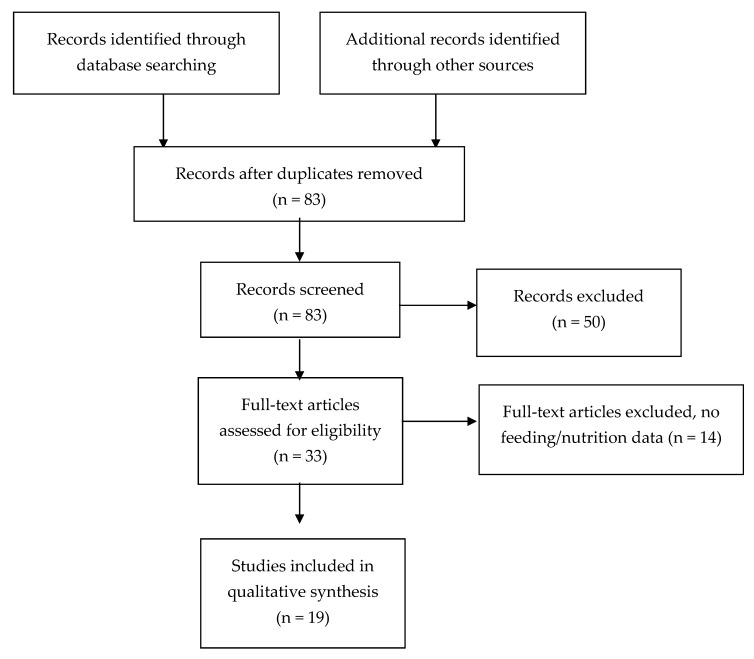
Flowchart of systematic review results.

Studies were included if pre- and post-operative characteristics of infants with SIP were provided, specifically if nutrition (enteral feeds prior to surgery and post-operative total parenteral nutrition (TPN)) data was recorded (Figure 1). Analysis of studies in languages other than English on non-human subjects and review articles were excluded. Case reports where no data on survival or length of hospital stay was reported were also excluded. 

### 2.3. Study Selection

All abstracts and titles identified using the search criteria were independently reviewed by the first two authors (O.O. and M.S.) and irrelevant studies were removed. The full text of relevant articles was reviewed by O.O. and M.S. for inclusion criteria until a consensus was reached. Screening reference lists was performed by O.O. 

### 2.4. Data Collection Process

Selected articles were classified by study type and divided into two groups based on reporting of nutrition data prior to SIP (enteral nutrition) and post-operatively. Variables extracted included patient demographics/characteristics (gestational age, weight) and feeding characteristics (route, type of feeding, timing). Outcomes extracted included length of stay (LOS), time to full enteral nutrition, duration of TPN and mortality/survival as well as long-term neurodevelopmental outcomes when reported. For the meta-analysis, retrospective cohort studies that reported timing of early enteral nutrition as well as relative risk of outcomes were included. 

## 3. Results

### 3.1. Inclusio

There were 33 full text articles that met criteria for full-text review. Of these, 14 were subsequently excluded because no information on enteral nutrition prior to SIP or TPN data post-operatively were reported. A total of 19 articles (Table 2, Table 3 and Table 4) were included in the analysis (Figure 1). Included articles are summarized according to category of nutritional data—studies with nutrition prior to SIP in Table 2; studies with data after surgery in Table 3; outcomes reported are outlined in Table 4. There were no randomized control trials (RCTs) in neonates examining the impact of early enteral nutrition on SIP progression and outcomes after surgery. However, 12 studies retrospective cohort studies (III-2), two studies with historical controls (III-3) and one case series [9] were included (Table 2 and Table 3).

### 3.2. Risk of Bias 

Most of the studies were retrospective cohort studies conducted at single centers, including Buchheit [4], Eicher [29], and Gollin [30] who reported characteristics and outcomes in infants with SIP and NEC. This study design has an inherent risk of selection bias, and inherent differences between the pathogenesis and complications associated with NEC can skew results, especially given the retrospective nature of these studies. Additionally, only two studies by Stavel et al. [24] and J Shah [3] et al. included data on control infants without NEC or SIP. There is a risk of detection bias as it is not possible to blind outcomes in these studies. 

Data from the case series by Meyer et al. [9] was confounded by potential information and reporting bias given the retrospective nature and lack of a control group. 

### 3.3. Grouping According to Nutrition Data

Articles fell broadly into three groups: studies that included feeding or nutrition data prior to SIP diagnosis [4,5,9,11,22,23,24], studies with data post-operative nutrition [3,16,21,26,29,30,31,32,33] and studies that listed any outcomes of interest (LOS, time to full feeds, length of TPN, mortality, neurodevelopmental outcomes; Table 4). 

Data on timing, type and volume of feeding prior to SIP was limited. In the study by Meyer et al. [9], no patients with SIP had been fed prior to disease onset. Two studies documented the proportion of patients receiving enteral nutrition (EN) before SIP: Buchheit [4] reported 38% (8/21) and Holland [11] 23% (6/23). Maas et al. [23] reported a rate of SIP of 9.4% after implementation of a feeding protocol for early transition to full EN. The only study evaluating the direct impact of early EN (within 72 h of life) on SIP was by Stavel et al. [24]. A total of eight additional studies [16,21,26,29,30,31,32,33] reported TPN data after surgery for SIP.

### 3.4. Outcomes

#### 3.4.1. Early Enteral Nutrition (EEN)

Varma [25] retrospectively reviewed the use of breast milk in infants who had surgery prior to six months of age. Eighteen out of 111 infants required surgery for SIP and 16/18 (89%) received human milk, and the median age at first feed was four (IQR 3–8) days. Maas et al. [23] described the feasibility of an EEN protocol in extremely low gestational age neonates (ELGANs, <28 weeks) with the initiation of 20 mL/kg/day of preterm formula or human milk within 24 h of life and advances of 25–30 mL/kg/day. Forty-three out of 96 (50%) infants received full EN by seven days of life. SIP was reported in 9/96 (9.4%) of infants. No data on timing of SIP was noted. While the incidence of SIP is comparable to similar European centers (8.2% by Bassler [34]), Maas did not report rates of SIP at their institution prior to initiation of this protocol. The study was not designed to evaluate the EEN as a protective factor for SIP but suggests that an EEN protocol is feasible in ELGANs. 

**Figure 2:**
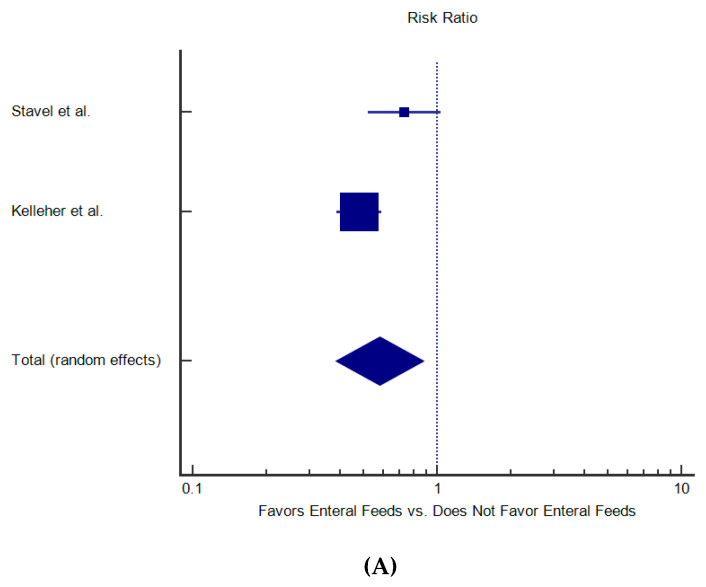

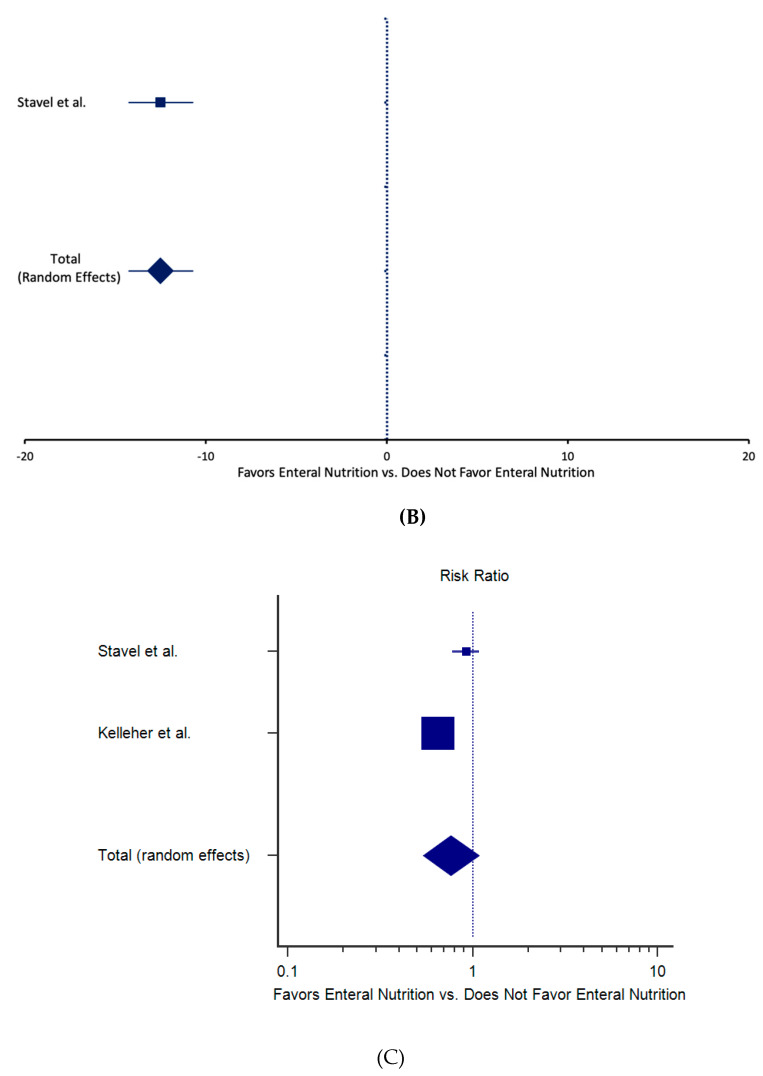
Relative risk and outcomes reported in selected studies. (**A**) Early Nutrition and SIP Incidence; (**B**) Early Nutrition and LOS; (**C**) Early Nutrition and overall mortality.

Stavel [24] from the Canadian Neonatal Network (CNN, tertiary NICUs) published a retrospective cohort study of 4268 ELBW infants born prior to 30 weeks evaluating the effect of exposure to prophylactic indomethacin and early feeding on the incidence of SIP [24]. EEN was initiated within the first two days [24]. There was a notable—although not significant—reduction in the incidence of SIP in the early feeding group (EF+, 54/2114, 2.5%) compared to the no EF group (EF-, 75/2154, 3.5%) with an adjusted odds ratio (aOR 1.32, 95% CI [0.88–1.99]) of SIP in the EF- group. However, there was no documentation about volume or type of EN provided. Kelleher from the Neonatal Research Network (NRN) performed a similar retrospective study of over 15,000 ELBW infants [21]. They reported data on four groups based on exposure to indomethacin (I+/−) and early feeding (first two days, E+/−). A significant reduction in relative risk (%, aRR [95%CI]) of SIP in the first 14 days of life was documented in the E+ groups (I+/E+: 3%, 0.58 [0.37–0.90] *p* < 0.05, I−/E+ 1%, 0.53 [0.36–0.77], *p* < 0.0001) compared to the reference group (I−/E−: 3%). Overall, when these studies were combined there was a significant reduction in relative risk of SIP in infants receiving early enteral nutrition (0.58 [0.38–0.88], *p* = 0.01, Figure 2A). Therefore, early enteral nutrition reduces the incidence of SIP in ELBW infants. 

#### 3.4.2. Time to Full Enteral Feeds after SIP

Eicher [29], Jakaitis [31], B Shah [32], Vongbhavit [26], Varma [25], Cass [27] and Wadhawan [33] reported time to initiation of EN (range from 6 to 21 days) and time to full EN (range from 15 to 95.5 days) after SIP (Table 3). Varma [25] reported days to first post-operative feed (median 12.5, IQR (10–20)) and the majority of SIP infants received human milk (16/18, 88%) and bolus feeding (15/18, 83%). Cass [27] reported shorter time from peritoneal drain (PD) placement to feeding initiation in infants with SIP (26.3 ± 9.9 days) compared to infants with NEC (73.5 ± 3.5 d *p* < 0.05). Eicher [29] reported the shortest time to initiation of EN with mean of six (range 4–9) days and full EN mean of 15 days after surgery. On the contrary, Jakaitis [31] documented the longest time to initiation of EN (20.1 days in peritoneal drain (PD) only, 26.1 days in PD and laparotomy (LAP) group, *p* < 0.05) and full EN (60.4 days in PD, 95.5 days in PD + LAP, *p* < 0.05). No defined protocol for initiation and advancement was described in either study. Vongbhavit [26] noted that differences in delay in initiation of post-operative EN increased the risk of parenteral nutrition-associated cholestasis (PNAC), defined as a conjugated bilirubin ≥ 2 mg/dL. Initiation of EN and full EN was shorter in SIP infants without PNAC (10 days, 25 days) compared to those with PNAC (20 days, 46 days *p* < 0.05). Institutional differences and type of surgery as reported by [31] resulted in variations in timing of EN. However, delay in initiation of EN increased the risk of PNAC. 

#### 3.4.3. Parenteral Nutrition Duration

Current management of SIP involves surgery (or drain placement), cessation of feeds, and a course of antibiotics [27,35]. Nutrition during recovery is exclusively provided by total parenteral nutrition (TPN), similar to infants being treated for NEC [36]. The most prevalent risk associated with prolonged TPN exposure is cholestasis [37]. Eleven studies ([21,24,25,26,28,29,30,31,32,36,38] Table 3) reported data on duration of TPN after surgery with a range in SIP patients of (21–94.3 days). TPN duration was shortest in Eicher [29], with an average of 21 days in infants with SIP likely due to early initiation of EN by six days post-op. Jakaitis [31] reported the longest duration of TPN use in the PD only (62.7 days) and PD + LAP group (94.3 days). This suggests that earlier initiation of EN appears to correlate with shorter duration of TPN. Stavel [24] evaluated the role of early indomethacin (I) and early feeding (E) on TPN duration. Number of days of TPN was shorter in both EN groups (I+/E+, mean(range): 18 (12–32) and I−/E+ 18 (12–29)) compared to no EN (I+/E− 28 (19–40, *p* < 0.01) and I−/E− 26 (16–39, *p* < 0.01)). EN initiated within the first two days of life decreased the duration of TPN in ELBW infants <30 weeks [24].

#### 3.4.4. Length of Stay

Eight studies [4,24,25,26,29,30,31,32] documented length of stay (LOS) in SIP and controls with a range from 72 days in I−/E+ group in the Stavel [24] study to 144.5 days in the PD + LAP group in the Jakaitis [31] study. In those studies where data on both NEC and SIP was available, no significant differences in LOS were noted in Buchheit [4] (82 days vs. 107 days), Eicher [29] (128 days vs. 121 days) and B Shah [32] (110 days vs. 98 days) in patients with SIP compared to NEC. Jakaitis [31] reported longest LOS with no significant differences between the two surgical options available, PD and PD + LAP (120.3 days vs 144.5 days). In Vongbhavit [26], patients without PNAC had significantly shorter LOS compared to the PNAC group (77 days vs. 123 days *p* < 0.05) likely due to earlier EN and shorter TPN duration. Stavel [24] reported shorter median (IQR) LOS in early feeding groups (I+/E+, 80 (50,118), I−/E+ 74 (45, 103)) when compared to no early feeding groups (I+/E− 99 (66, 124), I−/E− 86 (55, 112), Figure 2B) for all infants in the study. Data on the impact of EN on LOS in infants with SIP was not reported. 

#### 3.4.5. Neurodevelopmental Outcomes

Wadhawan et al. [33] retrospectively identified ELBW infants with SIP in the Neonatal Research Network database (1998–2005) and is the only study that reported neurodevelopmental impairment (NDI) in patients with SIP. NDI among survivors was defined as at least one of the following: cerebral palsy, bilateral blindness, bilateral deafness, Bayley Mental Developmental Index (MDI) or Psychomotor Development Index (PDI) less than 70 [33] at 18–22 months. Overall NDI among survivors was higher in SIP infants compared to no SIP (86/137 (62.8%) vs. 2614/7033 (37.2%) *p* < 0.0001). Similarly, MDI < 70 (72/134 (53.7%) vs. 2177/6953 (31.3%) *p* < 0.0001), PDI < 70 (65/133 (48.9%) vs. 1476/6892 (21.4%) *p* < 0.0001), and cerebral palsy (24/140 (17.1%) vs. 486/7418 (6.6%) *p* < 0.0001) were higher in infants with SIP [33]. However, these findings could be confounded by the increased incidence of severe IVH (grade 3 or 4) in patients with SIP (95/277 (34.3%) vs. 1942/11233 (17.3%) *p* < 0.05). The authors of the study did not perform a regression analysis that adjusted for potential confounders. Additionally, while age at first feeds was significantly later in infants with SIP (14.7 ± 15.1 vs. 7.4 ± 6.8 *p* < 0.05) no comparisons between feeding and outcomes were documented [33]. 

Kelleher [21] reported severe NDI at 18–22 months in four groups based on exposure to indomethacin (I+/−) and early (first two days) feeding (E+/−), with a significant reduction in severe NDI in E+ groups (I+/E+ %, aRR [95% CI] 25%, 0.72 [0.61-0.83], *p* < 0.0001, I−/E+ 20%, 0.76 [0.68–0.84], *p* < 0.0001) compared to the reference group I−/E− (34%) [33]. The median days (IQR) to full EN was also significantly shorter in the E+ groups (I+/E+: 19 (14,29) *p* < 0.001, I−/E+ 16 (11,25) *p* < 0.001) compared to the reference group (I−/E− 26 (18,38)) [21]. This suggests a protective effect of early nutrition on improved neurodevelopmental outcomes overall in ELBW infants; it is possible that this is similarly protective in infants with SIP. 

#### 3.4.6. Mortality

Mortality alone or in combination with NDI was reported in 17 studies (Table 4) [3,4,9,11,16,21,22,24,26,29,30,31,32] with a range of 8%–32% in infants with SIP. Shah [3] reported an almost three-fold increased mortality in infants with SIP compared to infants without NEC or SIP (aOR 2.78, 95% CI [1.8, 4.28] *p* < 0.05). Wadhawan [33] reported a significant and large increase in NDI or death in infants with SIP compared to ELBW infants without SIP (198/249 (79.5%) vs. 5568/9987 (55.8%) *p* < 0.001). 

However, mortality in ELBW infants is dependent on numerous factors, some of which can be modified by early enteral nutrition. Stavel et al. [24] and Kelleher [21] reported overall mortality in four groups based on one’s exposure to indomethacin (I) and/or early feeding (E) (Figure 2C). Stavel [24] noted a reduction in the overall mortality in the early feeding group (236/2114 (11.2%)) compared to the late feeding group (262/2154 (12.2%)) with an adjusted OR of 0.89 (95% CI [0.71, 1.12]). Although the reduction in mortality was not statistically significant, it suggests a possible protective effect of early enteral nutrition. In the Kelleher [21] study, there was a large and significant relative risk (RR) reduction in either death or NDI in the early feeding groups (I+/E+: 37%, aRR 0.83, 95% CI [0.75–0.91] *p* < 0.001, I−/E+: 31%, aRR 0.82, 95% CI [0.76–0.89], *p* < 0.0001) compared to the reference group (I−/E−: 48%). Overall, when these studies were combined, early enteral nutrition was not associated with increased mortality but rather has a trend towards decreased mortality (RR [95% CI], 0.764 [0.54–1.08], *p* = 0.13, Figure 2B). Thus, early feeding in the presence or absence of indomethacin is not associated with increased mortality. 

### 3.5. Other Complications

Shah [3] reported an increase (aOR) in major morbidity: bronchopulmonary dysplasia (2.78, 95% CI [1.93–4.20] *p* < 0.05), periventricular leukomalacia (1.62, 95% CI [0.85–3.07] NS), severe retinopathy of prematurity (3.14, 95% CI [1.88–5.2])) and nosocomial infections (3.54, 95% CI [2.54, 4.94] *p* < 0.05)) in infants with SIP vs. those without NEC or SIP (OR 4.23, 95% CI [2.88, 6.20], *p* < 0.05). No other studies reported on risk of short- and long-term complications relative to early nutrition or occurrence of SIP. 

## 4. Discussion

Although there are no randomized trials evaluating early nutrition in decreasing rates of SIP or SIP associated morbidities, the available data summarized in this review suggest that initiation of early enteral nutrition in ELBW infants decreases the incidence of SIP, duration of total parenteral nutrition, risk of parenteral nutrition-associated cholestasis and length of stay, all without being associated with increased mortality. Furthermore, evidence from a large cohort [21] suggests that ELBW infants receiving early enteral nutrition (with or without prophylactic indomethacin) have a lower incidence of neurodevelopmental impairment and mortality. Moreover, the overall growth improved in ELGANs who were fed using an early enteral nutrition protocol [23]. Consistent with existing data, introduction of early enteral nutrition using a standardized protocol has been associated with improved weight gain [39] and reduced incidence of NEC [40] and death.

There were limited data on initiating early enteral nutrition post-operatively in SIP patients, so optimal timing for initiation and impact of post-operative EEN remains unclear. However, data from a systematic review in pediatric patients (including neonates) undergoing abdominal surgery suggests that introduction of EEN post-operatively resulted in a significant decrease in time to full EN with a trend towards reduced LOS and no increase in complications [41]. 

**Figure 3:**
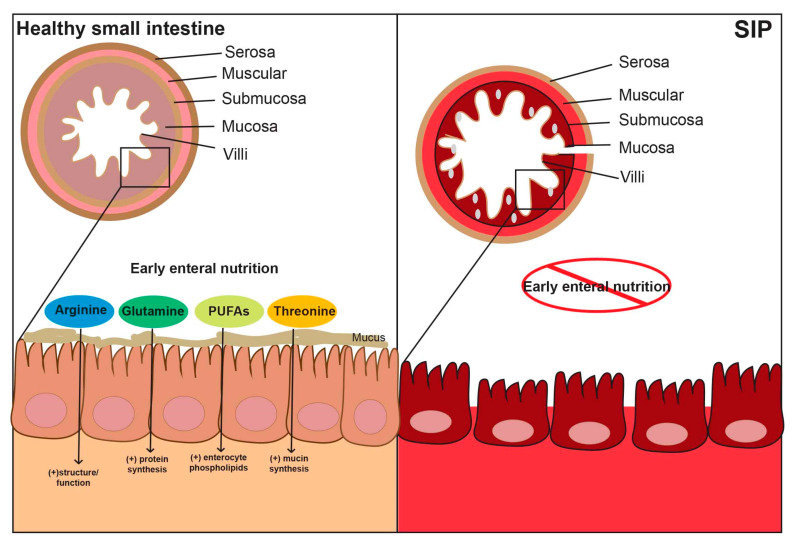
Relationship of early enteral nutrition to SIP. Early enteral nutrition provides arginine, threonine, glutamine and polyunsaturated fats (PUFAs) that result in improved gut structure/function, mucin synthesis and production of enterocyte phospholipids. Delayed enteral nutrition results in increased SIP susceptibility.

Timing and type of enteral nutrition provided after birth in neonates impacts intestinal health and immune function [42]. After delivery, enteral nutrition is crucial for intestinal adaptation, and lack of luminal nutrients can impede appropriate intestinal development (as reviewed in [42]). Enteral nutrition components that promote intestinal health include: (1) arginine, which improves structure and function, (2) glutamine for increased protein synthesis, (3) threonine, which promotes mucin synthesis, and (4) polyunsaturated fatty acids that enrich enterocyte phospholipids [42,43] (Figure 3). Early bovine colostrum feeds in animal models resulted in higher first-pass threonine utilization, increased protein synthesis and mucosal growth in the distal small intestine [43], as well as improved immune and digestive functions [44]. Similarly, in neonates who received human milk in the first 24 h of life, Shimizu et al. [45] reported an increase in plasma concentration of glicentin (a component of enteroglucagon that promotes mucin secretion and improved intestinal growth compared to delayed enteral nutrition). Human milk, specifically colostrum, is considered optimal enteral nutrition in preterm neonates as it results in decreased inflammatory response [46], stimulates neutrophil recruitment [44], selectively targets T cells and granulocyte [47], and resulted in reduction of SIP incidence (6% to 3%) in a small, single center study [48]. This suggests that in the absence of early enteral nutrition (as reported in [21,24]) there is likely reduced protein synthesis, decreased mucin production, impaired enterocyte phospholipids, inadequate mucosal growth and a predisposition to intestinal injury and subsequent SIP development. 

Currently, clinical studies do not provide adequate information on the timing or type of post-operative enteral nutrition in infants with SIP. However, in neonates who required surgery for congenital anomalies, post-operative early enteral nutrition resulted in decreased time to full enteral nutrition and a trend towards decreased hospital stays without increased complications [49,50,51]. Furthermore, post-operative implementation of a human milk-based feeding protocol resulted in reduced time to full feed and decreased incidence of intestinal failure-associated liver disease [52]. In animal models, post-operative early enteral nutrition resulted in improved healing [53], likely through conservation of collagen [54] and improved weight gain. It is possible that early initiation of enteral feeds after surgery in SIP patients is similarly beneficial, however additional studies are needed. 

## 5. Future Directions

Data from existing clinical studies show that early enteral nutrition in ELBW infants is feasible and beneficial. In the future, larger scale, multicenter studies dedicated specifically to patients with SIP evaluating the time to first feed, type of feeds (breast milk/formula) and feeding advancement schedules would be beneficial. In post-operative SIP cases, information regarding timing of first feeds as it relates to short-term outcomes (duration of hospital stay [44], parenteral nutrition dependence) and long-term outcomes (gastrointestinal complications, growth and neurodevelopment) would be important to collect. These data would inform us on best nutritional practices to reduce the incidence and severity of SIP.

## 6. Conclusions

The retrospective nature of studies that include the pre- and post-operative feeding and nutrition regimen of infants with SIP present a challenge in delineating the role of nutrition in disease prevention and improvement of outcomes. There is some evidence to suggest that initiation of feeds within the first 72 h in infants at the highest risk of SIP (ELBW, <28 weeks GA) could be protective. The potentially protective role of early feeding has been shown in both small and large retrospective studies [23,24,55,56]. 

## Figures and Tables

**Table 1:** Risk factors associated with increased incidence of SIP in preterm neonates.

Prenatal	Postnatal
Maternal preeclampsia	Medications
Chorioaminoitis	- Indomethacin
Syncytial knots	- Inotropes
Multiple gestation	- Early steroids
Cytomegalovirus	Fresh frozen plasma
*In utero* growth restriction	Intraventricular Hemorrhage

**Table 2:** Studies with documentation on feeding regimen prior to spontaneous intestinal perforation (SIP) diagnosis.

Authors	Institution(s), Country	Type of Study	Patients in Study (n)	Patients with SIP (n)	Mean GA (wks)	Feeding Regimen Prior to SIP	Comments
Buchheit [4]	University of Louisville, United States	R	42	21	29	Unknown	38% enteral feedings in the SIP, 86% in the NEC group (*p* < 0.005).
Kelleher [21]	Neonatal Research Network, United States	R	15751	652		Total Parenteral Nutrition ± Enteral Feeding	
Holland [11]	The Royal Alexandria Hospital for Children Australia	R	23	23	27	Enteral Formula Feeds	6 (26%) of the 23 patients received enteral feeds prior to development of SIP
Kawase [22]	Toho University Perinatal Center, Japan	R	556	10	26.3	Unknown	
Maas [23]	Tübingen University Children’s Hospital, Germany	R	77	9	26.7	Enteral feeds were initiated at 20 mL/kg/day of preterm formula on day 1.	Rates of NEC were low, whereas that of SIP was rather high at 9.4%.
Meyer [9]	Minneapolis Children’s Medical Center, United States	C	250	7		No enteral nutrition	
Shah, J [3]	The Canadian Neonatal Network, Canada	R	17426	178		Unknown	
Stavel [24]	The Canadian Neonatal Network, Canada	R	4268	129	SIP: 25 All: 34	DOL 0–2	
Varma [25]	Johns Hopkins University School of Medicine, United States	R	111	18		SIP (n = 18) Age at First Feed: 4 d. Mother’s Milk: 14 (78%) Donor’s Milk: 2 (11%) Cow’s Milk: 1 (6%) Hydrolysate: 0 Amino Acid: 0 Unknown: 1 (6%)	
	**Total:**		38504	1047			

R—retrospective chart review, C—case report, wks—weeks, GA—gestational age.

**Table 3:** Studies with documentation on post-operative nutrition in SIP patients.

Authors	Institution(s) Country	Type of Study	Patients in Study (n)	Patients with SIP (n)	GA (wks)	TPN Duration (after SIP)	Time to EN (Days)	Time to Full EN (Days)
Vongbhavit [26]	University of California at Davis, United States	R	60	30	PNAC: 25.5 Without PNAC: 25.9	Omegavan after 4 wks. w/DB > 2 mg/dL	PNAC: 20 Without PNAC: 10	PNAC: 46 Without PNAC: 25
Cass [27]	Texas Children’s Hospital, United States	R	21	10	SIP: 25.5 NEC: 27.5	Unknown	SIP: 26.3 NEC: 73.5	SIP: 41.6 NEC: 98
Chiu [28]	Children’s Memorial Hospital, United States	R	46	15	SIP: 26.7 NEC: 28.4	SIP: 24 NEC: 46	SIP: 16 NEC: 21	Unknown
Eicher [29]	Tübingen University Children’s Hospital in Tübingen, Germany	R	280	19	25	SIP: 21.0	SIP: 6	SIP: 15
Gollin [30]	Loma Linda University Children’s Hospital, United States	R	29	29	25.0 ± 1.5	68.8	Unknown	68.8
Jakaitis [31]	Children’s Healthcare of Atlanta at Egleston, United States	R	89	89	PD:25.1 PD + Lap: 25.8	PD: 62.7 PD + Lap: 94.3	PD: 20.1 PD + Lap: 26.1	PD: 60.4 PD + Lap: 25.9
Karila [16]	University of Helsinki Children’s Hospital and University of Tampere Children’s Hospital, Finland	R	225	83	27	Unknown	Unknown	Unknown
Kelleher [21]	Neonatal Research Network, United States	R	15751	652	I+E+: 26 I+E−: 25 I−E+: 27 I−E−: 26	I+E+: 19 I+E−: 28.5 I−E+: 17 I−E−: 29	Unknown	I+E+: 19 I+E−: 27 I−E+: 16 I−E−: 26
Shah B [32]	Women & Infants Hospital of Rhode Island, United States	CC	53	13	SIP: 25.8 NEC: 27.1 Control: 29.5	SIP: 76 NEC: 46 Control: 27	SIP: 10 NEC: 6 Control: 3	Unknown
Varma [25]	Johns Hopkins University School of Medicine, United States	R	111	18	SIP: 25 All: 34	SIP: 33.5 All: 51.5	SIP: 12.5 All: 12.5	Unknown
Wadhawan [33]	Neonatal Research Network, United States	R	11960	280	SIP: 26.3 No SIP: 26.9	SIP: 28.1 No SIP: 49.6	SIP: 14.7 No SIP: 7.3	Unknown
	**Total:**		28625	1238				

R—retrospective chart review, C—case report, CC—case control, wks—weeks, d—days, S.D.—standard deviation.

**Table 4:** Outcomes of studies that document feeding regimens prior to SIP diagnosis and those that document post-operative nutrition in SIP patients.

Authors		LOS (Days)	Enteral Feeds Prior to Perforation (Days)	Time to Begin Enteral Feeds (Days)	Time to Full Enteral Feeds (Days)	Length of TPN (Days)	Mortality	Risk of Bias
Buchheit [4]	SIP	82	8	X	X	X	5/21 (24%)	Low
	NEC	107	18	X	X	X	12/21 (57%)	
Cass [27]	SIP	X	3/10 (30%) *	26.3 *	41.6 *	X	1/10 (10%) *	Low
	NEC	X	10/11 (91%)	73.5	98	X	8/11 (73%)	
Chiu [28]	SIP	X	5/13 (38%) *	16 *	X	24 *	15% *	Low
	NEC	X	17/20 (85%)	21	X	46	45%	
Eicher [29]	SIP	128	X	6	15	21.0 *	3/19 (16%)	Low
	NEC	121	X	8	18	34.5 *	2/9 (22%)	
Gollin [30]	SIP & NEC	111	10/29 (34%)	X	68.8	68.8	38%	Low
Holland [11]	SIP	X	7/23 (30%)	X	X	X	26%	Moderate (convenience sample)
Jakaitis [31]	PD	120.3	36/67 (53.7%)	20.1 *	60.4*	62.7 *	18%	Moderate (criteria for groups unclear)
	PD + LAP	144.5	10/22 (45.5%)	26.1 *	95.5 *	94.3 *	5%	
Karila [16]	SIP	X	X	X	X	25	23%	Low
	NEC	X	X	X	X	27	27%	
Kawase [22]	Perf.	X	X	X	X	X	82/541 (15.2%)	Moderate (definition for groups unclear)
Kelleher [21]	I+E+	X	DOL 0–3	X	19 ^	19 ^	146/1185 (12%)	Low
	I+E−	X	X	X	27	28.5.	742/4674 (16%)	
	I−E+	X	DOL 0–3	X	16 ^	17 ^	287/3119 (9%)	
	I−E−	X	X	X	26	29	1037/6714 (16%)	
Maas [23]	ELGANs	90	96/96 (100%)	X	7	7	24%	Low
Meyer [9]	SIP	X	X	X	X	X	3/7 (43%)	Low
Pumberger [5]	SIP	X	13/13 (100%)	X	X	X	X	Low
	NEC	X	16/16 (100%)	X	X	X	X	
B. Shah [32]	SIP	110	100%	10 *	X	76 *	1/13 (8%)	Low
	NEC	98	100%	6 *	X	46 *	1/14 (7%)	
	Control	94	100%	3	X	27	2/26 (8%)	
J. Shah [3]	SIP	X	X	X	X	X	44/178 (24.7%)	Low
	NEC perf.	X	X	X	X	X	124/246 (50.4%)	
	NEC no perf.	X	X	X	X	X	101/538 (18.8%)	
	No NEC/perf.	X	X	X	X	X	902/16464 (5.5%)	
Vongbhavit [26]	PNAC	123 *	X	20 *	46 *	82 *	4/17 (24%)	Low
	w/o PNAC	77 *	X	10 *	25 *	32 *	14/43 (33%)	
Stavel [24]	I+E+	80^	DOL 0–2	X	23 ^	18 ^	35/285 (12.3%)	Low
	I+E−	99^	X	X	35 ^	28 ^	39/213 (18.3%)	
	I−E−	86^	X	X	29 ^	26 ^	223/1941 (11.5%)	
	I−E+	74	DOL 0–2	X	21	18	201/1829 (11.0%)	
Varma [25]	SIP	119.5 *	100%	12.5 *	17/18 (94%)	51.5 *	X	Low
	All	63	100%	10	103/111 (93%)	33.5 *	X	
Wadhawan [33]	SIP	X	X	14.7 *	X	48.1 *	198/249 (79.5%)* 5568/9987 (55.8%)*	Low
	No SIP	X	X	7.4 *	X	29.6 *	(NDI & Death)	

X: no available data, I: indomethacin, E: early feeding * *p* < 0.05, ^ *p* < 0.05 compared to reference group (I−/E−).
